# Smart Ambient Bright Light for Nursing Home Residents With Dementia: Neurobehavioral Symptom Impact

**DOI:** 10.1093/geroni/igaf122.1154

**Published:** 2025-12-31

**Authors:** Ying-Ling Jao, Diane Berish, Yo-Jen Liao, Julian Wang, Marie Boltz, Yun-Han Kuan, Light Ndurue, Margaret Calkins

**Affiliations:** The Pennsylvania State University, University Park, Pennsylvania, United States; The Pennsylvania State University, University Park, Pennsylvania, United States; The Pennsylvania State University, University Park, Pennsylvania, United States; The Pennsylvania State University, University Park, Pennsylvania, United States; The Pennsylvania State University, University Park, Pennsylvania, United States; Far Eastern Memorial Hospital, New Taipei City, Taipei, Taiwan (Republic of China); The Pennsylvania State University, University Park, Pennsylvania, United States; IDEAS Institute, Cleveland Heights, Ohio, United States

## Abstract

Most nursing home (NH) residents with dementia experience neurobehavioral symptoms. Lighting interventions have shown some positive impact on these symptoms, yet most interventions have not incorporated natural daylight. This study developed the Smart Ambient Bright Light (SABL) and evaluated its effect on neurobehavioral symptoms in nursing home residents with dementia. The SABL is an automatic lighting system that incorporates natural daylight and delivers bright light (high circadian stimulation [CS] and light intensity [lux] levels) during the day and dim light (low CS and lux) during the night. This 13-week crossover, cluster randomized controlled trial enrolled 27 residents with dementia and agitation in two Pennsylvania NHs. Neurobehavioral symptoms were measured biweekly using the Neuropsychiatry Inventory (NPI). Participants’ individual lighting exposures (CS and lux) were measured using an individual wearable light sensor clipped on their collar/shoulder all day, 7 days a week, every other week. Participants were 87.4 years old on average and primarily female (74%). Controlling for age, gender, and dementia stage, participants had significant reductions in appetite, depression, and nighttime behaviors during the intervention as compared to control periods. All other neurobehavioral symptoms also showed positive trends but did not reach significance. The lighting effect, based on individual lighting exposure, showed that higher daytime CS and intensity (lux) levels were significantly associated with lower apathy, appetite changes, delusions, and hallucinations. Individual CS levels were also significantly associated with fewer nighttime behaviors. Findings suggest that the SABL intervention has a positive effect on reducing neurobehavioral symptoms in NH residents with dementia.

